# Evaluation of the Impact of Selected Financial Indicators on Foreign Direct Investment in Bangladesh: A Nonlinear Modeling Approach

**DOI:** 10.1155/tswj/4406958

**Published:** 2025-04-18

**Authors:** Md. Sifat Ar Salan, Akher Ali, Ruhul Amin, Afroza Sultana, Mahabuba Naznin, Mohammad Alamgir Kabir, Md. Moyazzem Hossain

**Affiliations:** ^1^Department of Statistics and Data Science, Jahangirnagar University, Dhaka, Bangladesh; ^2^Department of Statistics, Mawlana Bhashani Science and Technology University, Tangail, Bangladesh

**Keywords:** financial modeling, foreign direct investment (FDI), generalized additive model (GAM), nonlinear modeling

## Abstract

**Background:** Foreign direct investment (FDI) is a steadfast contributor to capital flows and plays an indispensable role in driving economic advancement and emerging as a pivotal avenue for financing growth in Bangladesh. Therefore, this study identifies the factors that influence FDI inflows in Bangladesh. Moreover, the authors explored the more appropriate model for predicting FDI by comparing the efficacy of other models' predictions.

**Methods:** This study is based on secondary data over the period 1973 to 2021 and collected from the publicly accessible website of the World Bank. A generalized additive model (GAM) was implemented for describing the proper splines. The model's performance was assessed using the modified *R*-squared, the Bayesian information criterion (BIC), and the Akaike information criterion (AIC).

**Results:** Findings depict a significant nonlinear relationship between Bangladesh's FDI and key economic indicators, including GDP, trade openness, external debt, gross capital formation, gross national income (GNI) and government rates of exchange, total reserves, and total natural resource rent. It is also observed that the GAM (*R*^2^ = 0.987, *AIC* = 608.03, and *BIC* = 658.28) outperforms multiple linear regressions and polynomial regression in predicting FDI, emphasizing the superiority of GAM in capturing complex relationships and improving predictive accuracy.

**Conclusion:** A nonlinear relationship is observed between FDI along with the covariates considered in this study. The authors believed that this study's findings would assist in taking efficient initiatives for FDI management and proactive economic indicator optimization to empower Bangladesh's economic resilience and foster sustainable growth. The analysis revealed that FDI and its related risk factors follow a nonlinear pattern. The study recommends using the GAM regression as a reliable method for predicting FDI in Bangladesh. The authors suggest that the findings can guide policymakers in developing strategies to increase FDI inflows, stimulate economic growth, and ensure sustainable economic development in Bangladesh.

## 1. Introduction

Foreign direct investment (FDI) is a key driver of economic growth, especially in developing nations like Bangladesh. It serves as a critical source of capital, fostering technological advancements, job creation, and infrastructure development [1]. Bangladesh is on track to shift from a least developed country to a developing country which is a reflection of Bangladesh's commendable success in terms of key socioeconomic indicators of development [2]. Realizing sustainable development necessitates a committed investment in sustainability and environmentally friendly practices which is very important [3, 4]. Over the past few decades, FDI inflows in Bangladesh have increased due to favorable factors such as its strategic location, affordable labor force, and supportive economic policies. Insufficient investment results in depleted capital stock, diminished productivity, reduced output, and perpetuation of the cycle of poverty [4]. In today's financial landscape, FDI stands as the predominant and steadfast contributor to capital flows, playing an indispensable role in driving economic advancement and emerging as a pivotal avenue for financing the growth of a country [5]. Moreover, FDI is an essential cornerstone of investment creation and the potential sustenance over many developing countries with GDP, external debt stocks, inflation or GDP deflator, gross capital formation (GCF), total natural resources rents, gross national income (GNI), official exchange rate, total reserve (TR), and relative trade openness [6]. Despite this progress, understanding the underlying determinants of FDI remains challenging due to the complex and nonlinear relationships among various economic indicators.

In 2022, global FDI flows dropped by 24%, with the United States leading as the top destination, while China, Canada, Brazil, and India followed suit; in terms of outflows, the United States led, trailed by Germany, Japan, China, and the United Kingdom [7]. In contrast to other underdeveloped and developing countries, Bangladesh is experiencing a notable increase in FDI inflows. Foreign investment holds paramount importance in efficiently propelling the economy of an emerging nation like Bangladesh towards accelerated growth. The FDI stands as a vital form of foreign investment in Bangladesh, constituting a pivotal aspect of the nation's economic landscape. In 2021–2022, Bangladesh had a nationwide savings—a GDP percentage of 29.35% and an investment and a GDP level of 32.05%. Notably, those numbers changed from 30.22% to 31.25%, correspondingly, during 2022–2023, signaling dynamic shifts in the nation's economic landscape [8], indicating a decrease in the gap by 1.67% (from 2.70% to 1.03%). This trend may reflect the combined influence of FDI inflows and the escalating impact of domestic savings on the economy. Since its inception in 1980, FDI in Bangladesh has witnessed a substantial increase both in terms of volume and the breadth of sectors it operates within [9]. Bangladesh heavily relies on FDI to fuel its economic development, offering financial benefits, innovations, and employment prospects [10]. The nation's appeal to foreign investors lies in its competitive advantages such as low wage rates and stable economic policies [11]. However, factors like labor costs, market size, and economic openness influence FDI flows [12]. The research underscores the positive correlation between FDI inflows and factors like GDP, human resources, and governance quality, highlighting the importance of conducive economic environments [13]. Additionally, macroeconomic indicators like GDP and infrastructure development significantly impact FDI inflows [14]. Infrastructure investment, particularly in transportation and telecommunications, attracts foreign investors seeking efficient resource utilization and market access [15]. The FDI presents Bangladesh with opportunities to address socioeconomic challenges like poverty reduction and infrastructure development, integrating its economy into the global landscape [16].

Many researchers employed various methods to predict their chosen aspects. Nowadays, various nonlinear models, neural networks, and/or artificial algorithms are widely utilized in a variety of sectors for prediction, even though parameter understanding is challenging [17–24]. Traditional modeling approaches, including linear regression and autoregressive distributed lag (ARDL) models, have been extensively used to investigate these relationships. While these methods provide valuable insights, they often fall short of capturing the nonlinear and complex interactions inherent in economic data [14, 25]. However, the exploration of influential factors for FDI in Bangladesh has become a compelling subject for contemporary researchers, with limited studies employing nonparametric methodologies to examine this ability. Recent advancements in financial modeling, particularly the integration of AI-driven techniques and nonlinear econometric approaches, offer new possibilities for more accurate predictions. Studies have shown that machine learning algorithms, kernel regression, and nonparametric methods such as generalized additive models (GAMs) can effectively address the limitations of traditional models by capturing intricate patterns in the data [18, 24]. These techniques have proven successful in various sectors, including stock market prediction, trade forecasting, and economic growth modeling, yet their application to FDI analysis in Bangladesh remains limited. In this study, the authors utilize the GAM methodology, as a nonparametric approach, to analyze the evolving effects of significant economic indicators such as GDP, trade openness, external debt, GCF, GNI, official exchange rate, TRs, and total natural resource rent on FDI in Bangladesh to fulfill the research gap by identifying the complex as well as nonlinear pattern. This study provides valuable contributions to ongoing research and policy development in Bangladesh, offering insights into various aspects. Firstly, this study presents innovative discoveries stemming from an extensive nonparametric examination of the correlation complex relationship between FDI and influential factors, effectively filling the void in existing scholarly literature.

Secondly, this study breaks new ground by exploring the potential effects of GDP, trade openness, external debt, GCF, GNI, official exchange rate, TRs, and total natural resource rent on FDI in Bangladesh, marking a pioneering effort to understand the interplay between FDI and crucial economic metrics within the Bangladeshi context. Thirdly, this study utilizes the most recent and extensive dataset spanning 48 years (1973–2021) for its analysis. Fourthly, various modeling techniques, including GAM, polynomial regression (PR), and multiple linear regression (MLR), were examined to predict FDI outcomes. The prediction accuracy of the selected models used in this study was assessed through measures such as BIC, AIC, and *R*-squared. Finally, the results of this study can guide policymakers in formulating impactful strategies related to capital inflows, employment opportunities, technological transfer, market access, and economic growth. These strategies aim to bolster Bangladesh's economy sustainably by attracting increased FDI. The study's results are valuable for assessing financial tactics and devising innovative approaches to better equip Bangladesh for the global economy. This involves reinforcing actionable plans and policies that grasp the implications of economic stability, thus fostering long-term sustainable economic development. The findings drawn from this study could offer insights to developing nations seeking to fortify their economic development strategies towards exacerbation and rigidity, while concurrently devising effective plans to attain economic sustainability.

## 2. Methods and Materials

### 2.1. Data and Variables

Yearly time series data is collected from 1973 to 2021, including 10 variables. These variables included FDI in millions as the response and GDP (current $) in billions, external debt stocks (% of GNI) ($), inflation or GDP deflator (annual %), GCF (% of GDP), total natural resources rents (% of GDP), trade openness, GNI ($) in billions, official exchange rate (LCU per), and TRs (includes gold) ($) in billions as covariates. The secondary data were extracted from the World Bank's website [26] and accessible via the following link: https://data.worldbank.org/country/BD.

### 2.2. Study Design

**Figure 1:**
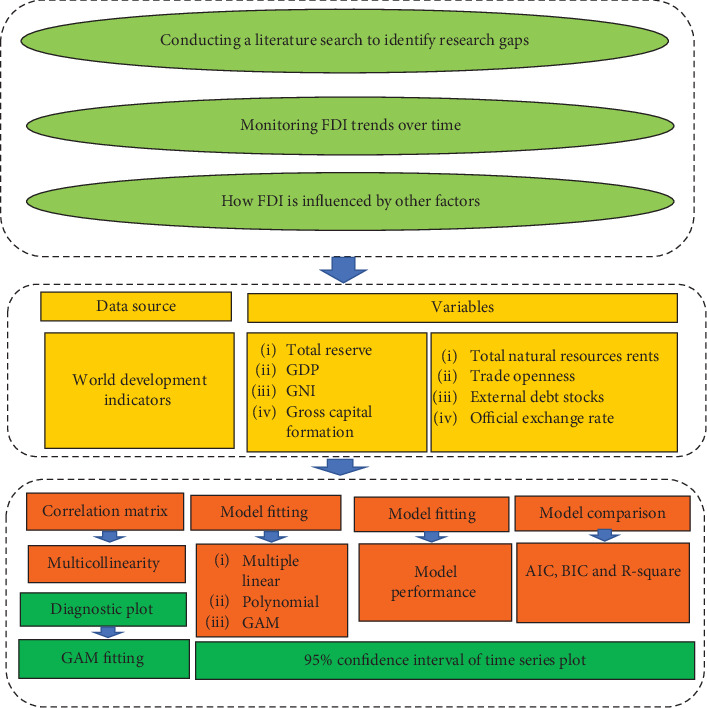
Framework of this study.

The meticulous selection of covariates stems from a rigorous examination of extant literature, meticulous data sourcing, and the profound self-assurance exhibited by the authors. This approach ensures that stakeholders, including governmental bodies and legislators, can strategically accumulate on the pivotal factors we have pinpointed as indispensable for fostering FDI in Bangladesh. By prioritizing these factors, informed decisions can be made to bolster the country's investment landscape and spur sustainable economic growth.  Figure 1 illustrates the conceptual framework adopted in this study. As an initial step, an exhaustive examination of the literature about the investigation of FDI is conducted to pinpoint any research gaps. Subsequently, the authors employed a plot over time to analyze the trajectory of FDI in Bangladesh. During phase two, the authors establish the data source and define the study variables. The ultimate stage entails evaluating model fitting, performance, and comparison through diagnostic assessment and generating predictions.

### 2.3. MLR Model (MLRM)

Under usual notations, the MLR equation can be written in the following way [27]:

 $$\begin{array}{c} FDI=\beta_{o}+\beta_{1}TR+\beta_{2}\;GDP+\beta_{3}GNI+\beta_{4}\;GCF+\beta_{5}\;TNRR+\beta_{6}\;\text{TO}+\beta_{7}\;EDS+\beta_{8}\;OER+\beta_{9}\;Inflation+\varepsilon . \end{array}$$

Here, *β*_*i*_; *i* = 1, 2, ⋯, 9 represent the parameter associated with the explanatory variables, and *ε* denotes the error term.

### 2.4. Assessment of Multicollinearity

Multicollinearity emerges in multiple regression models when multiple explanatory variables demonstrate significant linear correlation [28]. The variance inflation factor (VIF) is widely used as a means to detect multicollinearity problems and can be defined as

 $$\begin{array}{c} VIF=\frac{1}{1-R^{2}}=\frac{1}{Tol} \end{array}$$

where *Tol* represents tolerance. A VIF between 1 and 5 indicates moderate multicollinearity, while a VIF of 5 or greater indicates higher levels of multicollinearity [29]. Visualizations are also used to present the findings [30].

### 2.5. Nonlinear Regression

A relatively straightforward nonlinear framework is written in the following way:

 $$\begin{array}{c} Y=f\left(X,\beta \right)+\varepsilon \end{array}$$

where *X* represents the explanatory variable, *β* is the dimension of the parameter vector, *f*(.) is the previously established functioning, and *ε* is the variability of the error component [31].

### 2.6. PR

PR is an aspect of statistical analysis of regression in which the variable that is independent (*X*) in addition to the dependent variable (*Y*) has a nonlinear relationship that can be approximated by a polynomial function. In PR, the causal connection can be represented by an *n*th-degree polynomial expression. For instance, a simple form of PR with one independent variable (*X*) may have been expressed in the following way [32]:

 $$\begin{array}{c} Y=\beta_{o}+\beta_{1}x+\beta_{2}x^{2}+\beta_{3}x^{3}+\cdots +\beta_{n}x^{n}+\varepsilon . \end{array}$$

### 2.7. Piecewise Polynomial Spline

The equation that showcases a collection comprising every modular polynomial operation of degree *k* characterized by the break sequence *ξ* [33] is denoted here:

 $$\begin{array}{c} \prod <k,\xi . \end{array}$$

If we can allocate distinct cubic polynomials to four segments of *X*, delineated by the cutoffs {*C*_1_, *C*_2_, *C*_3_} [34], the fitted values can be obtained as

 $$\begin{array}{c} \hat{Y}=\left\{\begin{array}{c} \beta_{20}+\beta_{21}X+\beta_{22}X^{2}+\beta_{23}X^{3}\;\text{for }c_{1}\leq X<c_{2}\\ \beta_{30}+\beta_{31}X+\beta_{32}X^{2}+\beta_{33}X^{3}\;\text{for }c_{2}\leq X\leq c_{3}\\ \beta_{40}+\beta_{41}X+\beta_{42}X^{2}+\beta_{43}X^{3}\text{for }c_{4}\leq X \end{array}.\right. \end{array}$$

### 2.8. GAM

The conventional addition to general linear models is a GAM. A generalized linear model (GLM) with a linear predictor interacts with the total of smooth covariate functions, and it generalizes a general linear model by permitting the additivity of nonlinear functions of the variables [35]. The idea of GAM can be obtained by combining the additive model and GLM concepts as given below:

 $$\begin{array}{c} g_{i}\left(\mu_{Y}\right)=\sum f_{i}\left(X_{i}\right)=\beta_{0}+f_{1}\left(X_{1}\right)+f_{2}\left(X_{2}\right)+\cdots \end{array}$$

here, *Y* is the response variable, *X*_1_, *X*_2_, ⋯ are the covariates, and *f*_1_(∙), *f*_2_(∙), ⋯ are the unspecified and nonparametric smooth functions. The benefit of GAM is to reduce the error in predicting *Y*, from different distributions by evaluating general functions that are linked to the dependent variable through a link function. The functions *f*_*i*_ can have a stated parametric form, like a cubic polynomial or an unpenalized regression polygon of a variable of interest, or they can be given in a nonparametric or semiparametric manner. Occasionally, they can be approximated using nonparametric means as “smoothing functions” [36]. To fit GAMs, the R-function “gam” is used together with the appropriate codes and descriptions. The backfitting algorithm was used by the function “gam” to incorporate the various smoothing or fitting techniques. The package is loaded using the (mgcv) command in R. This study used “gam.check()” to further assess the model's appropriateness.

### 2.9. Reasons for Utilizing GAM

The GAM was chosen due to its ability to capture nonlinear relationships between FDI and key economic indicators, which linear models often overlook. Unlike kernel regression, GAM provides better interpretability by estimating smooth functions for each variable, making it easier to analyze individual effects [37]. It can also model both linear and nonlinear relationships simultaneously, offering greater flexibility. While ARDL models are suitable for exploring short- and long-term relationships in stationary data, GAM is more effective for handling the nonlinear patterns in this dataset [38]. Moreover, GAM's diagnostic tools improve model evaluation, and its nonparametric nature reduces multicollinearity, resulting in more robust and reliable outcomes. These features made GAM the ideal choice for this study.

## 3. Results

The authors conducted a descriptive analysis and examined time series plots to gain initial insights into the characteristics of the variables used in this study. A comparative analysis is also undertaken to determine the more appropriate predictive model.

Table 1 presents descriptive details regarding the selected variables. Across the 48 years of FDI data, the standard deviation is 875.17, indicating considerable volatility. The GDP (in current US dollars) varied from 416.26 to 8.09 billion, with an overall mean of 88.17 billion and a standard deviation of 104.84 billion. The highest observed value for external debt stocks (% of GNI) ($) is 44.48, while the average annual inflation or GDP deflator stands at 10.42. GCF (% of GDP) fluctuates from a low of 6.15 to a peak of 32.21 dollars, with a mean value of 5.148 dollars and a standard error of 6.181 dollars over the study period. Total natural resources rents (% of GDP) exhibit the minimum standard error, while FDI shows the highest relative to additional variables (Table 1).

**Figure 2:**
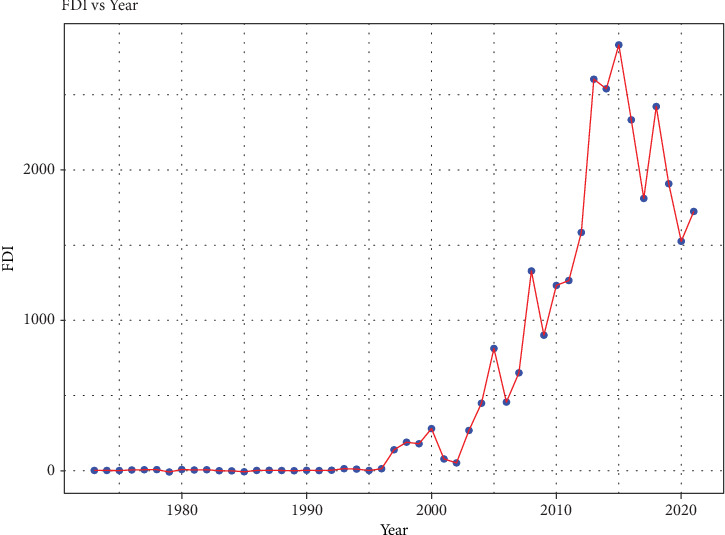
Time series plot of foreign direct investment (FDI).

Figure 2 illustrates that Bangladesh's FDI experienced a gradual increase over the years, particularly since 2001, reaching its peak in 2021 at 1723.86 million US dollars. Notably, between 2003 and 2011, there was a pronounced upward trend, followed by a period of relative stability before another upward trend emerged, persisting until the end of the study period.

**Figure 3:**
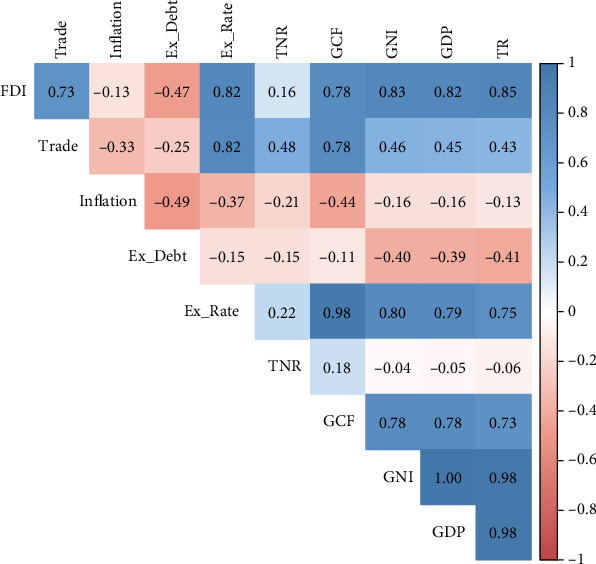
Correlation matrix plot.

According to Figure 3, FDI exhibits a positive association among several variables, except for inflation and external debt (Ex_Debt), indicating that all of these factors rise the FDI in Bangladesh. Findings indicate a positive relationship between FDI and TRs, with a correlation coefficient of 0.85, suggesting that higher TRs tend to attract more FDI. Similarly, value added GNI, with a correlation value of 0.83, also shows a strong positive association with FDI, indicating that an increase in value added GNI leads to higher FDI.

Moreover, GDP and FDI demonstrate a substantial positive association, with an associated coefficient of 0.82, implying that nations with higher GDP tend to attract more FDI. The exchange rate (Ex_Rate) also displays a positive association with FDI, with a coefficient of 0.82, suggesting that countries with stronger exchange rates tend to attract more FDI. Additionally, GCF and trade exhibit positive relationships with FDI, with correlation coefficients of 0.78 and 0.73, respectively. Conversely, inflation and external debt show negative correlations with FDI, indicating that countries with higher external debt tend to minimize their FDI (Figure 3).

A linear model is capable of being utilized to forecast the variable of interest based on the measurements of the explanatory variables, as well as to verify claims regarding their connection. They may additionally be employed to determine whether modifications in the covariates affect the response variable. The results of MLR are presented in Table 2.

**Figure 4:**
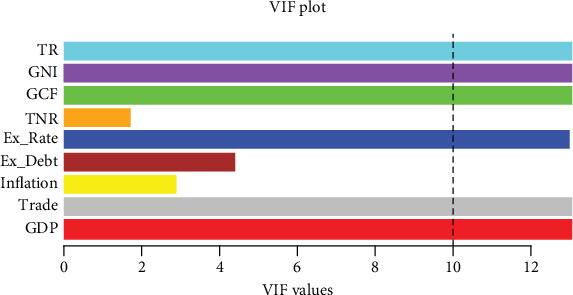
Plots of the results of VIF of the selected variables.

The variable TR is statistically significant, as shown with *p*-values. This indicates that these factors are significant determinants of the dependent factor in the regression model. The variables GDP, trade, inflation, external debt, exchange rate, GCF, resource rent, and GNI are not statistically significant at a 5% level of significance (*p* > 0.05). This means that there is inadequate proof to show that these factors are relevant. The VIF values vary between 1.72 and 11719.53, which suggests multicollinearity. To detect multicollinearity, the authors created a plot presenting the values of VIF, which is illustrated in Figure 4. The plot shows that GDP, trade, exchange rate, GCF, GNI, and TR have VIF values of greater than 10, indicating strong multicollinearity within these variables as well as the other variables that are independent in the model of regression [39].

**Figure 5:**
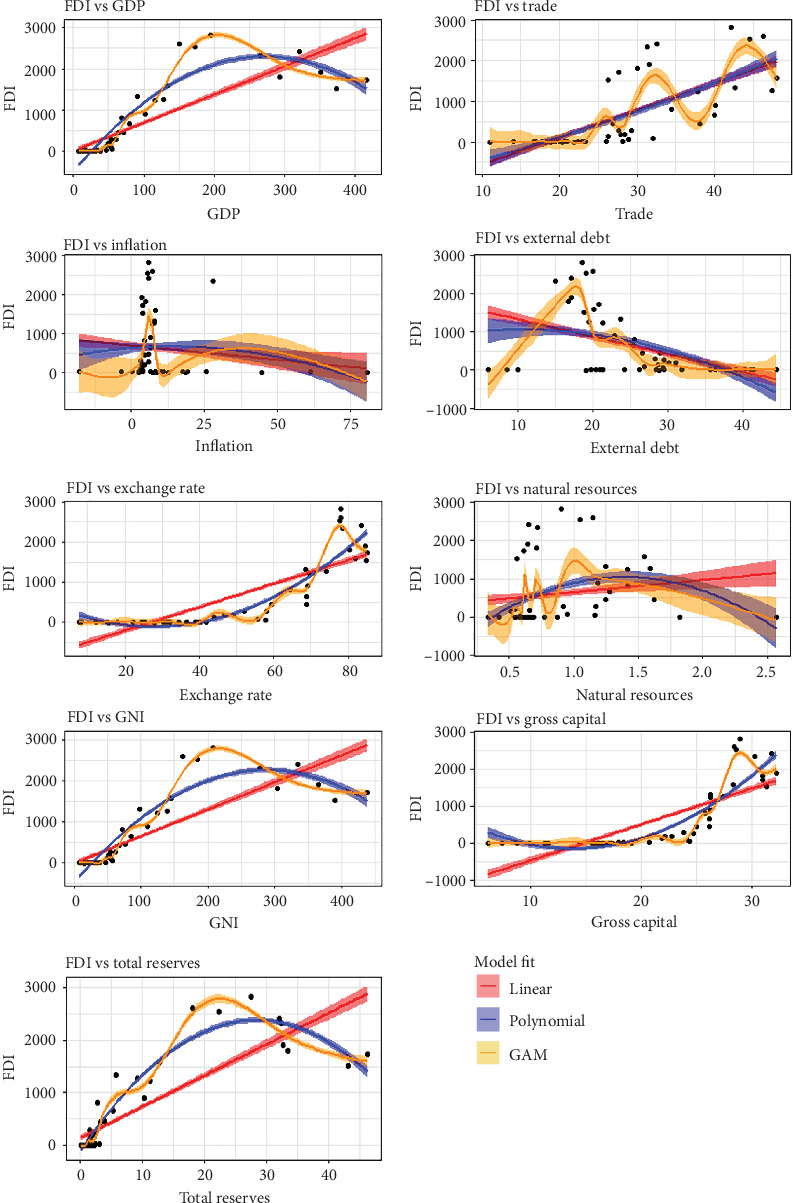
Linear and nonlinear plots of FDI against selective covariates.

These could result in incorrect parameter estimations and decreased model performance because a linear model is not appropriate for the available data. In contrast, multicollinearity can be dealt with in a variety of methods while conducting regression analysis. To address multicollinearity, the authors first use line and scatter plots to look for a straight-line connection between the one being studied in the study's outcome and the remainder of the components that were chosen.  Figure 5 displays scatter plots alongside 95% confidence intervals for three models of the considered variables along with the FDI.

Figure 5 shows that all covariates that are not linear correlated to the experimental variables, indicating complicated interactions between covariates that cannot be captured by a model with a linear relationship [40]. A nonlinear or nonparametric approach can be more suitable for capturing complex relationships. Nonparametric models offer a unique advantage by not imposing specific functional assumptions on the relationship between variables, granting them unparalleled flexibility. Conversely, nonlinear models excel in capturing complex nonlinearities within the data. Leveraging nonlinear or nonparametric modeling techniques on our dataset holds the promise of effectively tackling the underlying issue. By employing these advanced methodologies, it can be scrutinized whether the response of each variable to the dependent variable exhibits nonlinearity.  Figure 5 demonstrates that numerous covariates are amenable to nonlinear and nonparametric regression models, validating their efficacy for our dataset. Further, the authors conduct quadratic PR analysis without presuming associations within the dataset, followed by rigorous hypothesis testing to ascertain the statistical significance of coefficients within the quadratic PR model. The authors consider the following hypothesis.

*H*_0_ : *β*_1_ = *β*_2_ = ⋯ = *β*_12_, which showed that a quadratic polynomial model of regression is not significant.

*H*_*A*_ : *β*_1_ ≠ *β*_2_ ≠ ⋯≠*β*_12_, which showed that a quadratic polynomial model of regression is significant.

The results indicate that all variables including GDP, trade, external debt, exchange rate, and GNI exhibit small *p*-values (all below 0.05), implying their substantial contribution to explaining variations in the response variable. Moreover, the high *F*-values relative to their degrees of freedom for each source of variability offer robust evidence of their statistical significance. These findings, given in Table 3, underscore the considerable influence of each variable in elucidating variations within the dataset.

PR is prone to being influenced by outliers and can lead to overfitting, whereas GAM is less affected by outliers and does not tend to overfit as readily. Hence, it becomes evident that an ideal model for the dataset would integrate covariates based on their specific associations, blending both linear and nonlinear relationships within the explanatory variables. Hence, the authors chose to utilize the GAM for our analysis, with comprehensive outcomes presented in Table 4. In the nonlinear component, only total FDI and inflation were determined to be insignificant, while the remaining variables exhibited significance. Consequently, this model effectively addresses the multicollinearity concern present in the linear model. The first column of Table 3 represents the estimated values of the covariates use in this study. Assessing the curvature's degree of nonlinearity is indicated by the effective degrees of freedom (edf), a pivotal measure applied extensively in GAM analysis [36]. An edf value of 1 signifies a linear relationship, between 1 and 2 indicates a weakly nonlinear relationship, and values greater than 2 imply a highly nonlinear relationship with the variable. The juxtaposition of edf against reference degrees of freedom serves as a fundamental gauge of the table's goodness of fit.

The visual depiction of degrees of freedom further reinforces this evaluation. The majority of edfs closely align with the reference degrees of freedom, with several being equal, as evident in both the table and figure. This alignment strongly suggests the adequacy of the fitted model (Table 4).

**Figure 6:**
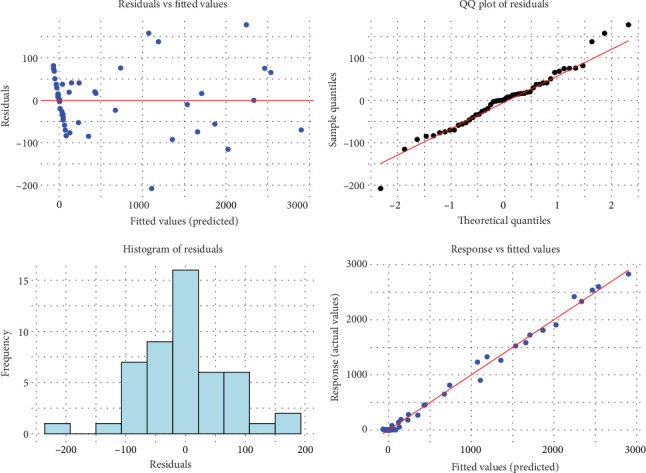
Visualization depicting diagnostic checks for the fitted GAM.

At the outset, it is imperative to thoroughly examine the foundational dimensions utilized for smooth terms to ensure they are not arbitrary defaults that might induce excessive smoothing. Moreover, the fitting process may not consistently handle deviations from distributional assumptions as effectively as a standard GLM, warranting additional caution. The susceptibility of smoothness selection via restricted maximum likelihood (REML) and maximum likelihood (ML) to departures from the assumed response distribution remains ambiguous. For instance, the smoothness selection criterion aims to minimize the scale parameter towards the specified one, potentially resulting in overfitting due to unmodeled overdispersion. To mitigate these concerns, an enhanced residual quantile–quantile (QQ) plot is employed. Wood proposes a methodology in 2017 to evaluate the appropriateness of the basis dimension for a smooth by estimating residual variance through comparing residuals with neighboring values in terms of the smooth's numerical variables [36]. Each visualization offers a unique perspective on the residuals of the model.  Figure 6 illustrates the outcomes of the initial model, which was insufficiently fitted. In one quadrant, a QQ plot contrasts model residuals with a normal distribution. Ideally, a well-fitted model would produce residuals that align linearly; however, this plot diverges from that ideal. Another quadrant displays a residual histogram, which ideally would be symmetric and bell-shaped, a characteristic our model roughly achieves. In a different quadrant, residual values should ideally exhibit symmetry around 0, yet ours are scattered. Lastly, a comparison of the response versus fitted values should ideally form a straight line, although absolute perfection is not expected. The authors anticipate the pattern to approximate the 1-to-1 line. Overall, the histogram's near bell-shaped appearance, the straightening QQ plot, and the closer clustering of response versus fitted values around the 1-to-1 line collectively suggest a significantly improved model fit (Figure 6).

**Figure 7:**
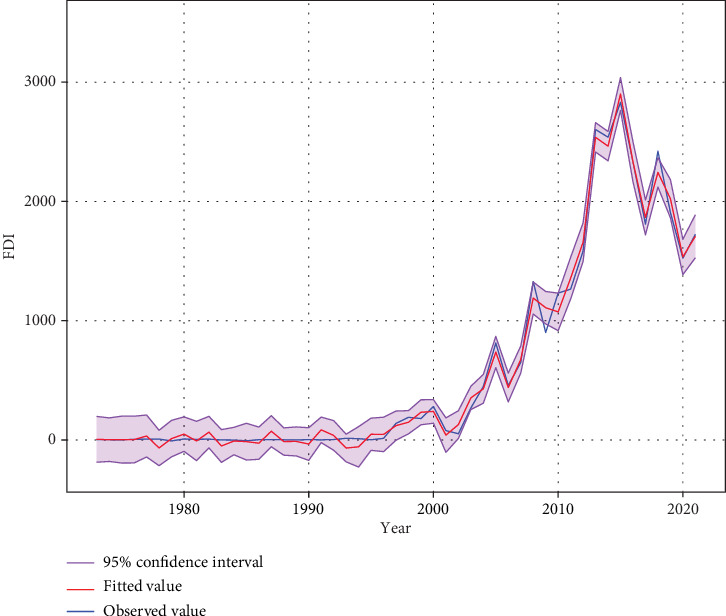
Prediction with 95% confidence intervals of FDI in Bangladesh by GAM.

Ultimately, to ascertain the optimal regression model, comparisons were conducted among three models: the quadratic PR, MLR, and the GAM, utilizing AIC and BIC criteria. The analysis described that the classical model yielded AIC 702.62 and BIC 723.43, while the quadratic polynomial model yielded AIC 664.17 and BIC 723.43. Conversely, the nonlinear GAM exhibited lower AIC (608.03) and BIC (658.28) values. This suggests a superior fit for our dataset with the GAM according to both AIC and BIC metrics.  Figure 7 presents a juxtaposition of observed and predicted values for Bangladesh's TRs derived from the GAM, along with a 95% confidence interval. Impressively, there is a negligible disparity between the observed and projected values, highlighting the exceptional alignment of the model with the dataset. For a more profound understanding of FDI trends and to evaluate the GAM's efficacy, the authors present the findings in a time series plot accompanied by a 95% confidence interval. The fitted line illustrates the expected values derived from the model fitted to the dataset, whereas the observed data line portrays the actual values across time. The confidence interval indicates both the model's uncertainty and the inherent variability in the data and delineates the span within which the authors can confidently deduce the true values lie, with a confidence level of 95%. Notably, the majority of fitted values fall within the confidence interval, bolstering our confidence in the GAM's ability to provide reliable estimations (Figure 7).

## 4. Discussion

The key objective of this study was to discern the key factors influencing FDI in Bangladesh using a yearly dataset extracted from the website of the World Bank. However, accomplishing this objective proved difficult without a suitable framework. Nevertheless, after noticing nonlinear trends in the data, the authors chose a model capable of accurately identifying the factors at play and clarifying the connection between the explanatory variables and the response. Thus, the authors decided to implement the GAM. Long-term FDI is notably and positively impacted by various factors, including GDP, trade volume, exchange rates, GCF, resource revenue, GNI, and reserves, and also negatively related to inflation and external debt. The research suggests that in Bangladesh, enhancements in trade, reserves, GDP, GCF, and resource revenue correlate with improvements in economic sustainability. Recent studies emphasize that trade openness, infrastructure development, and exchange rate stability are key drivers of FDI in South Asia. For instance, India's focus on IT and service sectors has significantly boosted FDI [41, 42], while Sri Lanka's improved infrastructure has attracted investments in manufacturing and logistics [43]. A cross-country analysis highlights market size, labor force growth, and trade policies as critical factors for increasing FDI inflows in developing Asian countries, including Bangladesh [44]. Compared to India and Sri Lanka, Bangladesh's reliance on manufacturing and energy sectors offers an opportunity for diversification into high-value sectors like technology and renewable energy [45]. The findings align with prior studies conducted in Bangladesh [46–52], which confirmed the positive correlation between GDP, exchange rates, trade openness, reserves, revenue, and economic expansion. Moreover, this study investigates the potential of Bangladesh to foster a sustainable economy through the utilization of GDP, trade, and reserves. The results indicate that enhancing influential factor sources while augmenting overall revenue can potentially reduce the reliance on FDI in Bangladesh. This is because factors such as GDP, trade volume, GCF, GNI, and reserves have been found to exert a notable and positive impact on FDI. This conclusion is in line with earlier research [53–57].

Based on the findings of this research, traditional trade practices need an enhancement through the integration of modern technology to bolster the economy, attract FDI, and secure reserves for Bangladesh. Many global organizations like the World Trade Organization (WTO), the International Monetary Fund (IMF), and the World Bank have developed intelligent trade strategies to adjust and improve trade expansion, aiming to reduce possible economic harm. These strategies include various measures such as diversifying trade, reducing tariffs, facilitating investments, and providing financial support for trade. Through customization of trade policies to tackle emerging issues and risks, these entities aim to strengthen economic resilience and foster sustainable development amid the dynamic global economic scenario [58, 59]. These findings will significantly contribute to fostering sustained economic growth and fostering global economic transformation. To optimize FDI inflows, Bangladesh must enhance trade practices, infrastructure, and technology integration while improving fiscal policies to control inflation and external debt. Modern trade strategies by the WTO, IMF, and the World Bank, such as trade diversification, tariff reduction, and investment facilitation, could strengthen economic resilience. Tailored trade policies can help Bangladesh mitigate risks and capitalize on high-value sectors like IT and renewable energy. Through better governance, infrastructure development, and sustainable trade policies, Bangladesh can reduce its reliance on FDI in traditional sectors while fostering long-term economic growth and global competitiveness.

### 4.1. Strengths and Limitations

The research is assessing nonlinear as well as linear models to forecast FDI in Bangladesh, which introduces a unique aspect to existing literature. It is important to note that FDI can be affected by additional factors beyond the scope of this study. Furthermore, future research could explore alternative techniques such as ARDL and kernel regression models for comparison with the GAM utilized here. Moreover, harnessing cutting-edge machine learning and artificial intelligence algorithms has the potential to greatly augment future research endeavors in this field.

### 4.2. Policy Implications and Recommendations

The study's findings highlight key areas where targeted policy interventions can boost FDI inflows and foster sustainable growth in Bangladesh. Policymakers should prioritize introducing tax incentives for foreign investors, particularly in high-potential sectors like renewable energy, ICT, and manufacturing. Simplifying trade regulations, reducing bureaucratic delays, and enhancing customs efficiency can create a more favorable business environment. Strengthening the financial and legal framework such as ensuring transparent regulations, stable exchange rates, and reliable contract enforcement will reduce investment risks. Additionally, infrastructure development through public–private partnerships (PPPs) and investments in human capital are crucial for attracting high-tech industries. Finally, sector-specific policies promoting green technologies and the digital economy can align FDI with national development goals. A phased roadmap focusing on short-term measures (tax incentives and regulatory simplification), medium-term infrastructure upgrades, and long-term institutional reforms will help ensure sustainable FDI growth.

## 5. Conclusion

The researchers employed the GAM, a nonparametric approach, to capture the observed nonlinear relationships between FDI and key predictors. To ensure robustness, three regression models MLR, quadratic PR, and GAM were initially tested for their predictive performance. The results demonstrated that GAM outperformed the other models, effectively capturing complex patterns in the data and providing superior predictive accuracy for FDI in Bangladesh. Beyond its methodological contributions, this study underscores the importance of incorporating flexible, nonlinear modeling techniques in economic forecasting. Future research could extend these findings by integrating AI-driven predictive models, exploring sector-specific FDI trends, or examining the interplay between policy reforms and investment flows. By providing a more accurate and dynamic framework for FDI analysis, this research can inform policymakers in designing data-driven strategies to enhance investment attractiveness, economic resilience, and sustainable growth in Bangladesh. The researchers sought to employ a nonparametric technique (GAM) to apply a nonlinear model, guided by visual observations suggesting robust nonlinear associations among the response variable (FDI) and the predictors. In this study, three regression models were initially utilized to predict FDI. Among the models (MLR, quadratic PR, and GAM) used to examine the prediction performance, the GAM exhibited superior performance in predicting the FDI in Bangladesh.

## Figures and Tables

**Table 1:** Descriptive statistics of the selected variables.

**Variable**	**Min**	**Max**	**Average**	**Standard deviation**
FDI (response variable) in million	−8.01	2831.15	605.18	875.17
GDP (current $) in billion	8.09	416.26	88.17	104.84
External debt stocks (% of GNI) ($)	6.14	44.48	25.98	8.98
Inflation or GDP deflator (annual %)	−17.63	80.57	10.42	15.40
Gross capital formation (% of GDP)	6.15	32.21	21.00	7.06
Total natural resources rents (% of GDP)	0.34	2.57	0.89	0.43
Trade openness	11.00	48.11	27.41	9.48
GNI ($) in billion	8.11	438.17	92.40	109.99
Official exchange rate (LCU per)	7.85	85.08	47.75	24.70
Total reserves (includes gold) ($) in billion	0.14	46.17	7.85	12.45

**Table 2:** Results of the multiple linear model.

**Variable**	**Estimate**	**Std. error**	*p * ** -value**	**VIF**
(Intercept)	−316.02	548.70	0.57	—
GDP	−27.02	41.17	0.52	11,283.38
Trade openness	30.11	16.37	0.07	14.61
Inflation	1.37	4.49	0.76	2.89
External debt	−13.61	10	0.18	4.40
Exchange rate	7.37	11.13	0.51	45.77
Gross capital formation	2.57	31.60	0.94	30.15
Total natural resource rent	−70.14	122.71	0.57	1.72
GNI	18.08	39.99	0.94	11,719.53
Total reserves	100.58	21.89	<0.001^∗∗∗^	44.97

⁣^∗∗∗^*p* < 0.001.

**Table 3:** ANOVA results for the quadratic polynomial regression model test.

**Variable**	**Sum of squares**	**Df**	**Mean square**	*F * ** -value**	*p * ** -value**
GDP^2^	3.25 × 10^7^	2	1.62 × 10^7^	495.61	<0.001^∗∗∗^
Trade^2^	1.08 × 10^6^	2	5.44 × 10^5^	16.58	<0.001^∗∗∗^
Ex_debt^2^	2.25 × 10^5^	2	1.12 × 10^5^	3.43	<0.05^∗^
Ex_Rate^2^	8.54 × 10^5^	2	4.27 × 10^5^	13.02	<0.001^∗∗∗^
Inflation^2^	1.21 × 10^4^	2	6.06 × 10^3^	0.18	0.83
TNR	5.54 × 10^4^	1	5.54 × 10^4^	1.69	0.20
GCF^2^	3.12 × 10^3^	2	1.55 × 10^3^	0.05	0.95
GNI^2^	8.54 × 10^5^	2	4.27 × 10^5^	13.00	<0.001^∗∗∗^
TR^2^	1.15 × 10^5^	2	5.76 × 10^4^	1.76	0.19
Residuals	1.02 × 10^6^	31	3.28 × 10^4^	—	—
Performance indicator	*R*-squared = 0.967, AIC = 664.17, BIC = 700.12				

⁣^∗∗∗^*p* < 0.001, ⁣^∗∗^0.01 < *p* < 0.05, ⁣^∗^0.05 < *p* < 0.1.

**Table 4:** The results of GAM.

**The estimated significance of linear components**					
**Coefficients**	**Estimate**	**Std. error**		*t * ** value**	*p * ** -value**
Intercept	605.18	16.01		37.81	<0.001^∗∗∗^

**The indicative significance of smooth components**					
	**Estimates**	**Effective degrees of freedom**	**Reference degrees of freedom**	*F*	*p * ** -value**
s(GDP)	9916.09	4.35	4.79	15.76	<0.001^∗∗∗^
	4461.78				
	5348.21				
	−19,343.01				
	−89,164.56				

s(Trade)	51.17	1.00	1.00	4.47	<0.05^∗^
	243.06				
	466.26				

s(Inflation)	33.46	2.80	2.96	1.21	0.28
	171.53				
	113.07				

s(Ex_Ddebt)	535.76	1.00	1.00	3.63	<0.01^∗∗∗^
	688.11				

s(Ex_Rate)	271.56	2.64	2.93	9.63	<0.001^∗∗∗^
	−1784.44				
	−3911.72				

s(GCF)	−771.72	3.00	3.00	11.99	<0.001^∗^
	518.21				
	6951.62				

s(GNI)	−28,910.83	1.00	1.00	43.52	<0.001^∗∗∗^
	80,833.76				

s(TNR)	3.16	1.00	1.00	4.50	<0.05^∗^
	−8.17				
	−8.01				
	−102.33				
	−265.41				

S(TR)	−195.23	5.00	5.00	11.49	<0.001^∗∗∗^
	−229.91				
	−338.54				
	−677.96				
	2848.56				

Performance indicator	*R*-squared = 0.987; AIC = 608.03; BIC = 658.28				

⁣^∗∗∗^*p* < 0.001, ⁣^∗∗^0.01 < *p* < 0.05, ⁣^∗^0.05 < *p* < 0.1.

## Data Availability

The data that support the findings of this study are available in the World Bank Database at https://data.worldbank.org/country/BD. These data were derived from the following resources available in the public domain: World Bank Database, https://data.worldbank.org/country/BD.
